# Personality traits and meat consumption: The mediating role of animal-related ethical concerns

**DOI:** 10.3389/fpsyg.2022.995482

**Published:** 2023-01-04

**Authors:** Gonzalo Haefner, Janosch Schobin, Antje Risius

**Affiliations:** ^1^Institute of Economics, Unit Empirical Economic Research, University of Kassel, Kassel, Germany; ^2^Department of Agricultural Economics and Rural Development, University of Göttingen, Göttingen, Germany

**Keywords:** animal ethical concerns, differential meat consumption, personality traits, animal ethics and welfare, big five personality

## Abstract

Prior research suggests that personality traits are associated with meat consumption. However, this association is not uniform across all types of meat. For instance, Big Five personality traits such as openness and agreeableness are negatively associated with red meat consumption but positively associated with fish. Using a large sample of Chilean university students (*N* = 1,149), we examined whether these differential meat consumption patterns can be explained by an intermediary variable of animal-related ethical values. Structural equation modeling was employed to test the hypothesized associations. The results suggest that animal-related ethical values mediate the effect of certain personality traits on the consumption of beef and poultry.

## Introduction

Environmental problems related to a steady increase in meat consumption have become one of the most pressing global environmental issues (Gardner, 2005; Steg and Vlek, 2009; Koger, 2010). To keep global warming below 2°C, a plethora of research suggests that a shift toward diets with a lower carbon footprint, especially plant-based diets, is necessary (Girod et al., 2014; Verain et al., 2015; Bryngelsson et al., 2016; Godfray et al., 2018). The increase in meat consumption also is noted to impact quality of life through human health outcomes (Micha et al., 2010; Bouvard et al., 2015; Domingo and Nadal, 2017), and to trigger concerns about the correct ethical treatment of animals involved in agricultural and livestock production (Cornish et al., 2016).

In some respects, the goal of more plant-based diets is increasingly out of reach (Howes et al., 2017). Latin America’s emerging middle classes have been aligning their lifestyles with those of the United States and European middle classes (Khara et al., 2020). This has led to a significant increase in high emission private consumption behaviors such as meat-based diets in demographically expanding populations (Williams and Anderson, 2020). Chile, where our study was situated, is a perfect example of this dynamic: Historical research indicates that meat consumption passed from annual consumption of around 30 kg per person of meat in the 1930s to over 90 kg per person in the 2010s. This increase has been especially stark since the 1990s (Llorca-Jaña et al., 2020). The Chilean case epitomizes that it is essential for the deployment of effective global environmental policies to investigate human dietary behavior in emerging economies. However, most studies on environmentally sustainable diets have been conducted in Western industrial countries.

Diverse research focusing on the interplay of psychological and social factors that influence meat consumption is therefore valuable to advance a broad range of sustainable development goals. Along with sociodemographic factors such as age, gender, income, and educational level (Lea and Worsley, 2001; Gifford and Nilsson, 2014) and attitudes toward meat consumption (Dhont and Hodson, 2014; Piazza et al., 2015; Monteiro et al., 2017), recent research has suggested that animal-related ethical concerns (Hölker et al., 2019a,b) and personality traits play an active role concerning whether or not and how often meat is consumed (Keller and Siegrist, 2015; Pfeiler and Egloff, 2018b, 2020).

In the present study, we extended this literature by examining if and how individual differences in the Big Five personality traits (McCrae et al., 2018) and animal-related ethical concerns (Hölker et al., 2019a,b) are interrelated and associated with different types of meat consumption in a Chilean sample. The main objective of the present study was to investigate a potential mediating role of animal-related ethical concerns between personality traits and different types of meat consumption. This aspect is still missing in the current debate. While there are existing studies dealing with the association between animal welfare ethics and meat-based diets (e.g., Schulze et al., 2018; Ohlau et al., 2022) as well as studies dealing with the association of personality traits and meat consumption (e.g., Keller and Siegrist, 2015; Pfeiler and Egloff, 2018b, 2020), empirical research on personality traits that combines both perspectives is missing. This is, in part, because studies that investigate how personality traits are connected to animal-related ethical values are still very rare. We herein contribute some of the first empirical evidence on this subject. Furthermore, since this study is the first of its kind to be carried out in a Latin American country, we aimed to assess if the antecedent role of personality traits on meat consumption that has been established in previous samples of Western industrial societies can be reproduced in the Chilean context.

In summary, the present study tries to shed light on the understanding of meat consumption by contributing novel evidence to the two ongoing but for the most separate debates that relate personality and animal-ethical values to meat consumption by combining both views in one study.

## Theoretical background

### Animal-related ethical concerns and meat consumption

Animal-related ethical concerns are among the most cited motivations for a reduced or meatless diet in Western culture (Ruby, 2012). Recent studies have suggested that omnivores’ beliefs concerning the ethics of meat-eating are a strong predictor of their meat consumption, whereas environmental and health-related determinants are much less predictive (Roozen and Raedts, 2022). Leveraging animal-related ethical concerns to promote meat-reduced diets still presents several difficulties, however. For instance, animal-related ethical concerns cannot be assumed to be homogeneously and consistently present across populations (Alonso et al., 2020). The concerns also tend to vary according to the species considered. The attribution of sentience, for instance, is often stronger for domestic animals, such as cats and dogs, than it is for livestock, fish, and insects (Hellyer et al., 1999; Hölker et al., 2019a). Moreover, beliefs about animal welfare ethics cannot be reduced to a single unidimensional construct.

Recent studies have revealed that these inconsistencies and complexities can be assessed more accurately when animal-related ethical concerns are considered through the lens of domain-specific values rather than through attitudinal information (Hölker et al., 2019a). Attitudinal information is often limited to a specific object (Eagly and Chaiken, 2005), whereas domain-specific values are strongly embedded in individual value systems and do not change as quickly as attitudes. At the same time, due to their concentration on a thematic complex, domain-specific values are relatively well-linked to specific consumer behaviors. This means that domain-specific values should offer a better prognostic quality and permit a higher degree of generalizability (see Hölker et al., 2019a). Nevertheless, research such as De Backer and Hudders’ (2015) study that uses domain-specific values as a predictive approach for meat consumption is still scarce.

A notable exemption is a seminal study carried out by Hölker et al. (2019a). It reveals that the consumer’s domain-specific animal-ethical values can be captured by seven distinct but correlated dimensions. These range from original anthropocentrism (“Humans are allowed to rule over animals as they please”) and anthropocentrism with indirect duties (“Humans are allowed to treat animals as they please, however, without cruelty, in order to avoid brutalizing humans”), through relationism (“Duties towards animals depend on their relationship to humans``) and utilitarianism (“Positive consequences of animal use have to outweigh its negative consequences”), to new contractualism (“Humans are allowed to use animals but should guarantee them, in turn, a good life”), animal rights (“Animals, as sentient beings, have inalienable rights”), and abolitionism (“The use of animals for human purposes should be abolished completely” (Hölker et al., 2019a). This conceptual frame allows for a more fine-grained understanding of individual animal-related ethical values than traditional attitudinal measurements. For instance, the domains can capture how participants who score high on the dimension of “relationism” attribute moral status to animals depending on their relationship to humans. This specific dimension captures a tendency toward positive attitudes toward the exploitation of farm animals, such as cows and chicken, but negative attitudes toward the exploitation of companion animals, such as dogs. Hölker et al. (2019b) argue that, in particular, those dimensions of animal-ethical beliefs that are related to the overall rejection or acceptance of the exploitation of animals are causally relevant for meat consumption. For instance, individuals who score high on the dimension of “abolitionism” attribute inalienable rights to animals. They believe that animals, being sentient and thus akin to humans, should be free from all forms of exploitation. In contrast, individuals who score high on “original anthropocentrism” believe in human exceptionalism and the right to exploit all animal species for any human purpose (Hölker et al., 2019a). Both dimensions, for this reason, are related to the inclination to eat animal-based products (Hölker et al., 2019b).

Moreover, following this novel approach to measure animal-ethical values, results of a recent experimental intervention in Chile suggest that pro-animal-ethical values such as abolitionism are related to the differential patterns of meat consumptions (Schobin et al., 2022). When “nudged” by friendly-looking animal cartoon faces, very abolitionist individuals were more likely to reduce the choice of red meats but not of fish. This suggests that meatless or meat-restricted diets, at least in the Chilean context, are motivated by the interplay between domain-specific values and personal ideas of the traits that certain species share with humans (Santos and Booth, 1996; Kenyon and Barker, 1998; Kubberød et al., 2002).

To extend this literature, we proposed the following hypotheses:

*H1a*: Abolitionism is negatively associated with beef and poultry consumption, but unrelated to fish consumption.

*H1b*: Original anthropocentrism is positively associated with beef, poultry, and fish consumption.

### The Big five personality model and meat consumption

Personality traits are relatively stable individual characteristics through which patterns of behavior, belief, and emotion converge and manifest in different situations (Parks-Leduc et al., 2015). In personality psychology, there exist several commonly-used scales to assess an individuals’ personality traits such as the Minnesota Multiphasic Personality Inventory (MMPI-2; Butcher et al., 1990), the 16 Personality Factor Questionnaire (16PF; Cattell, 1989), and the Big Five Personality Inventory (BFPI; Roccas et al., 2002). In methodological terms, there is a considerable overlap between the models that underlie these instruments: They all consider that an individual’s personality is a latent multidimensional construct. They mostly differ in the precise delimitations between and the exact number of personality dimensions.

For the present research, we opted for the BFPI’s five-factor personality model to facilitate the comparison with previous research. The BFPI is the most used personality assessment instrument in general and also in studies that specifically relate meat consumption to personality traits. The five-factor model considers five major personality traits: openness, conscientiousness, extraversion, agreeableness, and neuroticism (Goldberg, 1990; McCrae and John, 1992; McCrae and Costa, 1997).^1^ According to McCrae et al. (2018), the five-factor theory underlying the BFPI “offers a framework for causal explanations in personality psychology” (p. 152), conceiving personality traits as “abstract potentials, hypothetical psychological features of the individual that, over time and in specific situations, come to be manifested in concrete realizations” (McCrae et al., 2018, p. 152). Consistent with this claim, previous research underscores that BFPI traits are important predictors of many health and eating habits (Mõttus et al., 2012, 2013; Pfeiler and Egloff, 2020). They have been frequently used to identify patterns in individual meat consumption (Mõttus et al., 2012, 2013; Keller and Siegrist, 2015).

For example, Mõttus et al. (2012, 2013) report that meat-based diets are associated with lower openness scores and higher neuroticism scores after controlling for sociodemographic factors. Similarly, in a random sample (*N* = 951) of a Swiss population, overall meat consumption was negatively associated with openness and agreeableness Keller and Siegrist (2015). Analogous results were found by Pfeiler and Egloff (2018a). They investigated the personality correlates to a single-item assessment of general meat consumption in two representative German samples (“How often do you eat meat, fish, poultry, or sausages?” and “On how many days per week have you usually eaten meat, including poultry and various meat products such as sausages?”). Overall meat consumption was negatively associated with openness and agreeableness in both samples and negatively associated with conscientiousness in the second sample (Pfeiler and Egloff, 2018a).

Although these studies provide consistent evidence that personality is correlated with meat consumption—with lower levels of openness and agreeableness being related to greater meat consumption—meat consumption was assessed therein on single global scales. This reduces the precision of the findings, primarily because participants must combine different meat categories, which may obscure specific variation related to personality (Pfeiler and Egloff, 2018b). To remedy this issue, a few studies have evaluated the consumption of different types of meat separately. A recent study by Pfeiler and Egloff (2018c), for instance, examined the differential correlational patterns between red meat consumption, processed meat consumption, poultry consumption, fish consumption, and BFPI traits. The researchers asked participants to rate the frequency with which they consumed each of these four types of meat (Pfeiler and Egloff, 2018c). The findings highlighted that the association between meat consumption and BFPI traits is not uniform. Openness, conscientiousness, and agreeableness were all negatively correlated with red meat and processed meat consumption. Extraversion was positively related to poultry consumption. Neuroticism was negatively associated with red meat, processed meat, and poultry consumption. However, all BFPI traits were positively associated with fish consumption (Pfeiler and Egloff, 2018c).

Since one objective of the present study was to investigate the antecedent role of BFPI traits on meat consumption, the following hypotheses were proposed:

*H2a*: Openness and agreeableness are negatively associated with beef consumption.

*H2b*: Openness and agreeableness are negatively associated with poultry consumption.

*H2c*: All personality traits are positively associated with fish consumption.

### Personality traits and animal-related ethical concerns

Although personality traits and personal values correspond to two different constructs, they have been found to have consistent and significant theoretical relationships (Parks-Leduc et al., 2015). However, published, peer-reviewed, and quantitative research that explains the relationship between personality traits and animal-ethical values is rare (Herzog and Mathews, 1997; Eckardt Erlanger and Tsytsarev, 2012). Most research in this area has focused on attitudinal data regarding experimentation on animals or animal slaughter. Therefore, the association between animal-related ethical values and personality traits is a novel research field, particularly regarding how the moral development of the human-animal relationship interacts with underlying personality dimensions in motivating individuals to consume less or more meat.

Previous attitudinal research has found some patterns in the relationship between animal welfare ethics and personality traits. Herzog and Mathews (1997) administrated the 16 Personality Factor Questionnaire (Cattell, 1989) and the Animal Attitude Scale (Herzog et al., 1991) to a small undergraduate student sample (*N* = 99). The results yielded weak correlations: sensitivity and imaginativeness, which may be associated with the BFPI traits of agreeableness and openness, were related to animal welfare attitudes (Herzog and Mathews, 1997). Next, Eckardt Erlanger and Tsytsarev (2012) examined empathy, BFPI traits, and attitudes toward non-human animals. High scores in empathic concern and personal distress were related to discomfort with animal cruelty. Additionally, individuals with higher levels of BFPI openness were more likely to oppose utilitarian uses of animals (i.e., animal experimentation, hunting, and slaughtering). In a similar study, Furnham et al. (2003) found that all BFPI traits except for conscientiousness were associated with a negative attitude toward animal experimentation. Agreeableness was the strongest predictor. The authors explain that agreeable individuals are more sensitive to pain in others (Furnham et al., 2003) and are more likely to have high empathy, specifically empathetic concern, which is the extent to which one suffers when others are distressed (Furnham et al., 2003).

The theoretical explanation for these results is that first, people with higher levels of agreeableness also have higher levels of empathy and are thus more likely to demonstrate benevolent attitudes toward animals. Second, people with higher levels of openness are more flexible in opting for harmless alternative methods in areas like animal production (Eckardt Erlanger and Tsytsarev, 2012). Despite the differences between attitudinal data and domain-specific values, this theoretical-empirical background allowed us to generate certain theoretical resemblances. We assumed that the domain-specific dimensions of “abolitionism” and “original anthropomorphism” are linked to certain attitudes about animal suffering and death, and, consequently, are also related to the specific BFPI traits of agreeableness and openness.

We, thus, proposed the following hypotheses regarding the relationship between BFPI traits and animal-related ethical domain-specific values:

*H3a*: Openness and agreeableness are positively associated with abolitionism.

*H3b*: Openness and agreeableness are negatively associated with original anthropocentrism.

### The mediating role of animal-related ethical concerns

Personality traits represent a broad range of individual differences that provide limited results across behavioral domains at the expense of specific predictive ability (Saucier and Goldberg, 2004). BFPI traits can adequately predict broad types of behaviors (e.g., environment and health) but not more specific behaviors in specific domains (e.g., consumption of different types of meat; Epstein, 1980). Studies using the BFPI traits to examine the relationship between personality and differential meat consumption have assessed reliable interindividual differences (Keller and Siegrist, 2015; Pfeiler and Egloff, 2018a,b). However, they have failed to explain why certain traits are associated with certain types of meat consumption and others are not. The present study proposes that one way to address this limitation is to include mediating mechanisms responsible for the personality-behavior link.

**Figure 1:**
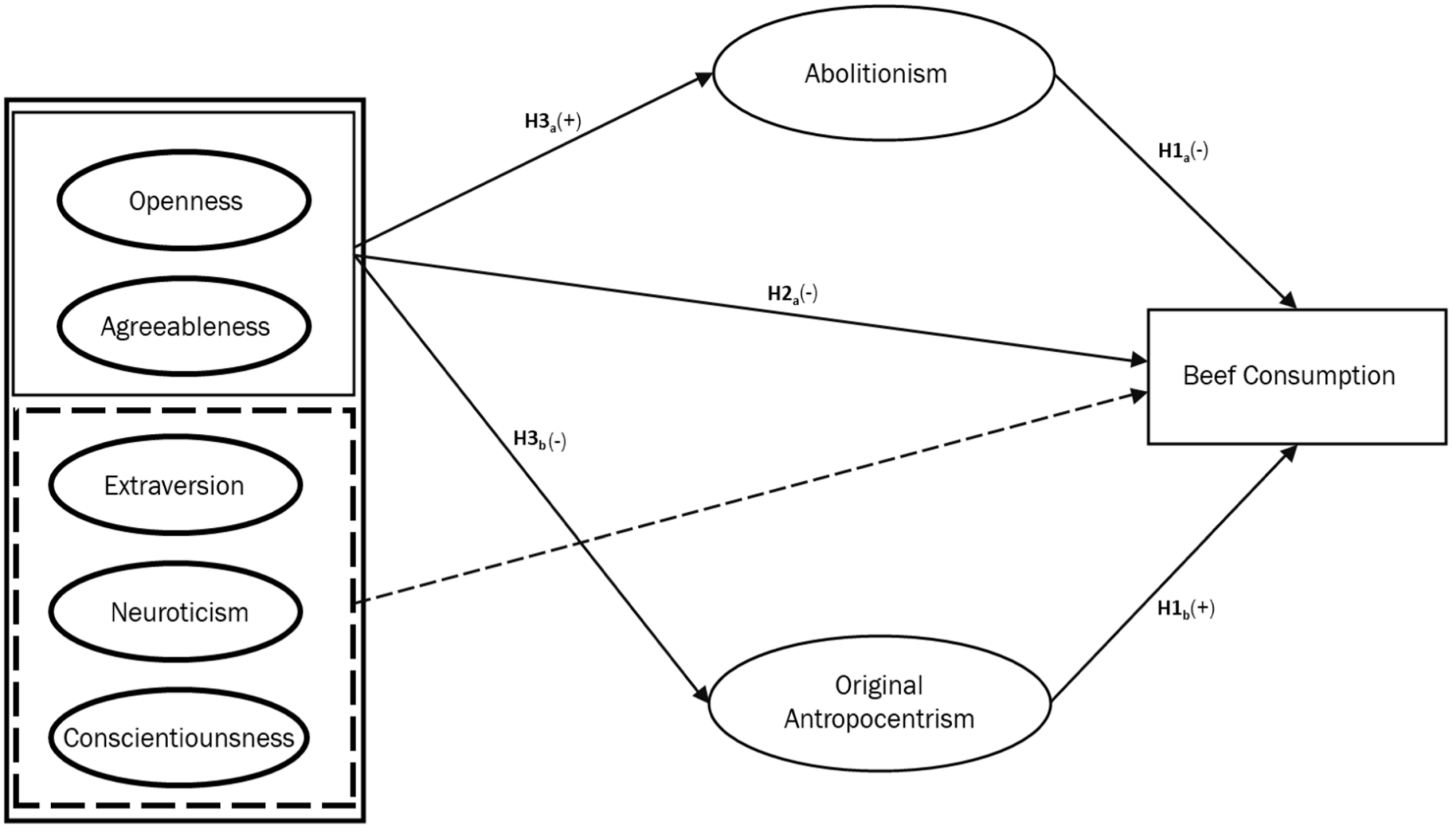
Conceptual model depicting the hypothesized relationships between the big five personality traits. Abolitionist ethical intuition, and frequency of beef consumption.

**Figure 2:**
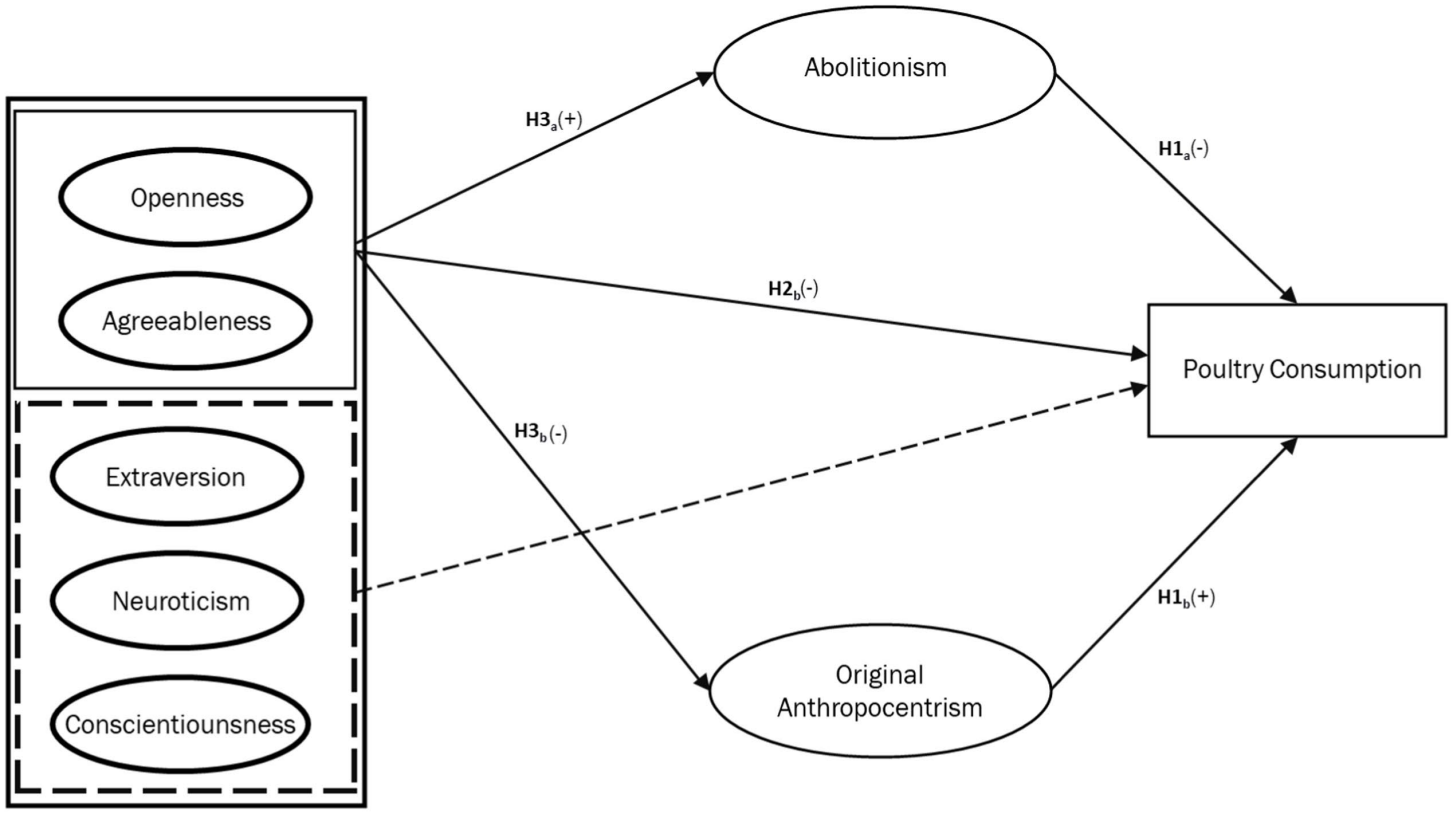
Conceptual model depicting the hypothesized relationships between the big five personality traits. Abolitionist ethical concern, and frequency of poultry consumption.

**Figure 3:**
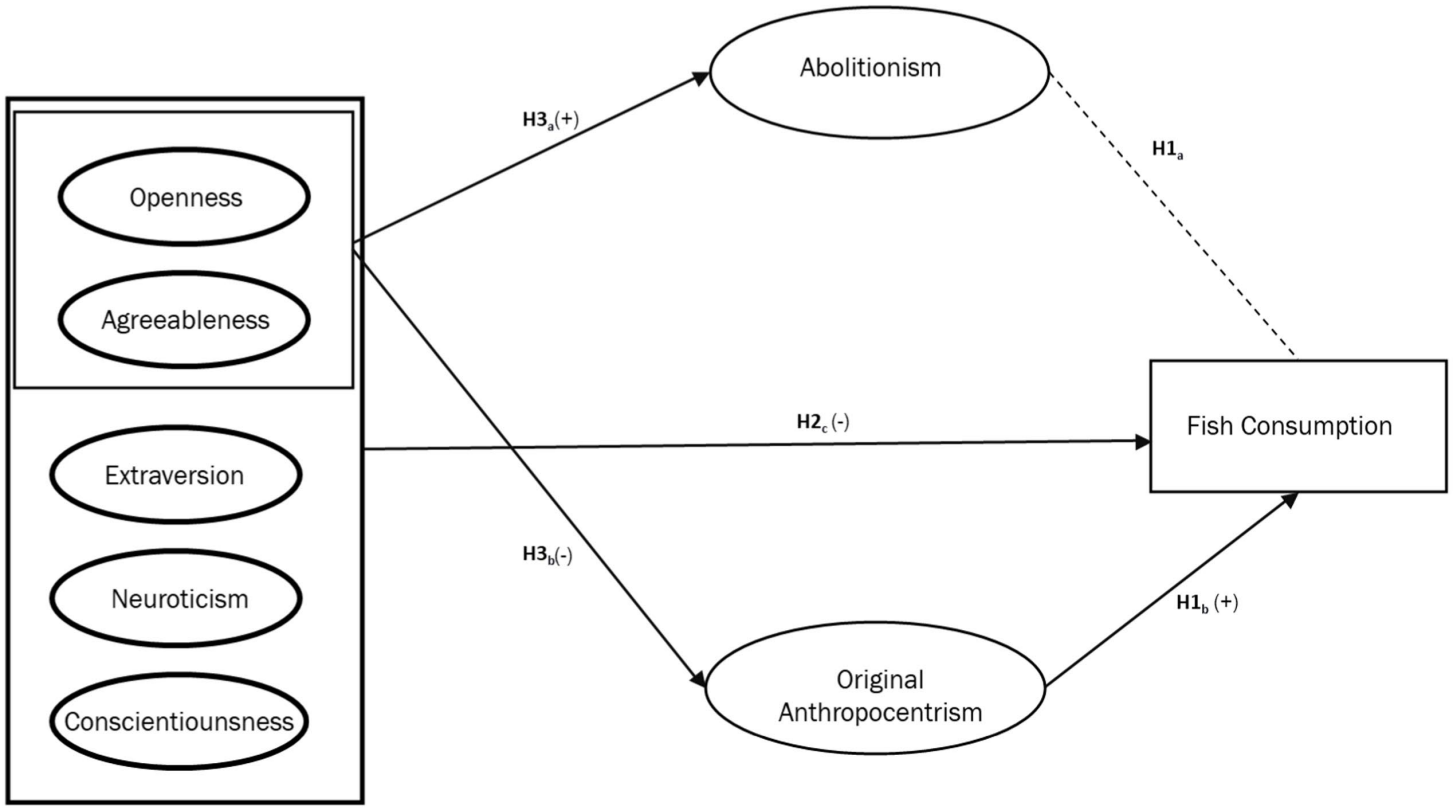
Conceptual model depicting the hypothesized relationships between the big five personality traits. Abolitionist ethical concern, and frequency of fish consumption.

As noted previously, personal values influence food choices (Graham and Abrahamse, 2017). Recent research has found that animal welfare ethics can be modeled as domain-specific values, with original anthropocentrism and abolitionism having significant direct effects on meat consumption (see Hölker et al., 2019a,b). However, the mediating role of these values in the relationship between personality traits and meat consumption has not previously been studied. We theorize that including such a mediating role could allow for more precise causal results. Abolitionism and original anthropocentrism are seen as negatively correlated but not mutually exclusive elements of the individual value system. The theoretical mediation model for this study thus conceptualized abolitionist and original anthropocentric ethical values as opposing mediators between BFPI traits and meat consumption, as seen in Figures 1–3, leading to the following hypotheses:

*H4a*: Abolitionist ethical values mediate the relationship between high openness and agreeableness and low consumption of beef, poultry, and fish.

*H4b*: Original anthropocentric ethical values mediate the relationship between low openness and agreeableness and high consumption of beef, poultry, and fish.

## Materials and methods

### Data collection and sample characteristics

Young, socially ascending individuals from the emerging economies of Latin America, Southeast Asia, and Africa are the most significant meat consumers of tomorrow (Sans and Combris, 2015). They are a growing demographic with a growing purchasing power. Understanding factors and dynamics that determine the meat consumption of university students in emerging economy contexts is, therefore, of central importance for the design of environmental policies. In the present study, we examined the association between personality traits, animal-related ethical concerns, and varied meat consumption in a large undergraduate university sample in Chile (*N* = 1,149).

### Measures

#### Sociodemographic information

The researchers analyzed gender (1 = male or diverse, 0 = female) and parents’ educational levels (1 = secondary education, 2 = tertiary education, 3 = Bachelor’s or equivalent level, 4 = Master’s or a higher degree level). Gender was recoded from three into two variables because the number of individuals who did not identify as either male or female was very low. This could lead to these individuals being identified, which must be avoided because in Chile, non-gender-conforming individuals suffer from high levels of minority stress. Because all participants were enrolled at the university, the researchers decided to use parental educational levels as a reliable proxy variable of the participants’ socioeconomic backgrounds. In Chile, educational level is tightly associated with social mobility and income. The participants thus provided the highest level of education attained by one of their parents.

#### Personality

Personality traits as constructs are generally more difficult to observe than demographic and socioeconomic information, yet they often have higher predictive power (O’Fallon and Butterfield, 2005). We concentrated on the standard personality measure of the BFPI. The traits of openness, conscientiousness, extraversion, agreeableness, and neuroticism were measured using a 21-item abbreviated scale based on Rammstedt and John (2005). After conducting confirmatory factor analysis, we dropped seven items for better fit (RMSEA = 0.054). The final 15-item scale consisted of questions rated on a 5-point Likert scale that ranged from 1 (“absolutely incorrect”) to 5 (“absolutely correct”). The Cronbach’s alphas were as follows: openness (*α* = 0.67), conscientiousness (*α* = 0.66), extraversion (*α* = 0.49), agreeableness (*α* = 0.59), and neuroticism (*α* = 0.73).

#### Meat consumption

This study used an abbreviated Food Frequency Questionnaire adapted from Thompson et al. (2002) to assess the participants’ consumption of four different meat-based products. Each question consisted of the following statement: “In a typical week, how frequently do you consume [beef; poultry; fish or shellfish]?.” The response levels were 1 = “Never,” 2 = “1 or 2 times a week,” 3 = “3 or 4 times a week,” and 4 = “5 times or more per week.”

#### Animal-related ethical concerns

The novel Animal Ethical Intuition (AEI) scale developed and validated by Hölker et al. (2019a,b) was administrated to assess the participants’ related domain-specific values. Hölker et al. (2019a) strove through the AEI scale to make a philosophical position useful for consumer studies, with two assumptions: First, ethical positions are based on complex argumentation schemes that cannot be captured entirely by a unidimensional construct. Second, society will not agree to animal-ethical positions without contradiction, meaning that respondents may advance more than one position. Thus, the AEI scale captures complex animal-related ethical concerns through seven correlated (nonorthogonal) dimensions. We translated and adapted the original AEI scale to the Chilean context and tested whether it had the same factor structure as in previous research (Schobin et al., 2022). The study was designed to focus on the two most extreme of the seven animal-ethical intuitions because the polarizing intuitions of original anthropocentrism (“Humans are allowed to rule over animals as they please”) and abolitionism (“The use of animals for human purposes should be abolished completely”) have been found to most significantly affect the consumption of animal-sourced foods (Hölker et al., 2019b). All six items were measured on a 5-point Likert scale from 1 = “strongly disagree” to 5 = “strongly agree.”

### Analytical procedures

Anderson and Gerbing’s (1988) two-stage procedure was followed for confirmatory factor analysis and structural equation modeling using RStudio (RStudio Team, Version 4.2.0, 2022) with the lavaan package version 0.6–11 (Rosseel, 2012). To assess the measurement model, we performed a confirmatory factor analysis that tested the relationships between items and their corresponding latent constructs. Average variance explained (AVE) and maximum shared variance (MSV) were administered to investigate the convergent and discriminant validity, respectively, whereas composite reliability (CR) was used to assess the internal consistency among scale items. According to Hair et al. (2013), AVE represents satisfactory convergent validity if > 0.5 and discriminant validity if AVE > MSV and CR > 0.7.

Moreover, we specified and compared two measurement models to evaluate the appropriate factor structure of the modified AEI scale. Sample size-independent model fit indices included root mean square error of approximation (RMSEA < 0.07), comparative fit index (CFI > 0.90), standardized root mean square residual (SRMR < 0.08), and the Tucker-Lewis Index (TLI > 0.90; Hair et al., 2013). We controlled for confounding effects of common method bias by applying an unmeasured latent method factor to the measurement model (i.e., gender and parental education level; Podsakoff et al., 2003). Finally, a bias-corrected bootstrap procedure with 5,000 resamples was run to test for any specific indirect effects of BFPI traits on meat consumption *via* abolitionism and original anthropocentrism.

## Results

### Reliability and validity of measures

To assess the appropriate factor structure of the AEI scale, we specified two measurement models, and their model fit was compared. The first model specified abolitionism and original anthropocentrism as a single unidimensional factor (immediate items reverse-coded). This model was underfitted: *χ*^2^(9) = 218.08, RMSEA = 0.14, CFI = 0.71, SRMR = 0.09, TLI = 0.51. The second model assumed, in accordance with prior research (Hölker et al., 2019b; Schobin et al., 2022), two correlated factors (i.e., abolitionism vs. original anthropocentrism). The model fit improved significantly: *χ*^2^(8) = 23.01, RMSEA = 0.04, CFI = 0.98, SRMR = 0.03, TLI = 0.96. Hence, a two-factor representation of the AEI scale was retained for further analysis.

The full measurement model included seven latent constructs: abolitionism, original anthropocentrism, openness, conscientiousness, extraversion, agreeableness, and neuroticism. The initial model fit was suboptimal: *χ*^2^(303) = 1610.46, RMSEA = 0.06, CFI = 0.75, SRMR = 0.062, TLI = 0.71. To improve the model fit, we screened the measurement model for problematic items (i.e., low factor loadings and high error correlations). Consequently, two items from three latent BFPI constructs—extraversion, conscientiousness, and neuroticism—were omitted. The final measurement model had a significantly improved fit: *χ*^2^(149) = 472.56, RMSEA = 0.04, CFI = 0.91, SRMR = 0.04, TLI = 0.88. Modification indices suggested some correlated error terms, which would improve model fit. However, allowing for correlated error terms—especially between items measuring different latent constructs—is usually not advised without a grounded theoretical reason (Landis et al., 2009; Hermida, 2015), and therefore, we did not implement such modifications.

Abolitionism and original anthropocentrism reported convergent validity (AVE > 0.50 and AVE > MSV). Both factors were below the CR threshold of 0.70. Openness, conscientiousness, agreeableness, and extraversion demonstrated adequate convergent validity (< 0.50) but were just below the CR limit (CR < 0.70). Neuroticism reported inadequate convergent validity (AVE = 0.44) and an appropriate CR value (> 0.70). The researchers considered it inappropriate to omit additional items to achieve a better overall fit and adequate validity and reliability estimates, based on the assumption that the latent constructs would only be reflected by one item and, hence, uncorrelated on their own (e.g., Howell et al., 2007). Table 1 presents the final measurement model.

**Table 1:** Standardized factor loadings, reliability, and validity.

Construct and item	Factor loadings	Composite reliability	Average extracted variance
*Openness*		0.69	0.69
I am interested in many kinds of things.	0.53		
I am intellectually curious and like to contemplate things.	0.51		
I am very imaginative.	0.61		
I enjoy artistic and aesthetic expressions.	0.63		
I do not have much artistic interest.	0.58		
*Neuroticism*		0.73	0.44
I get depressed or discouraged easily. I am laid back and do not let myself be worried about stress. I worry too much.	0.95 + +		
I easily become insecure and nervous.	0.91		
*Agreeableness*		0.51	0.68
I tend to criticize others. I trust another easily and believe that people are inherently good.	0.53 +		
I can be cold and distant in my behavior.	0.40		
I can be rude and devaluing to others.	0.75		
*Conscientiousness* I strive to finish my homework well.	+	0.54	0.63
I make things comfortable for myself and tend to be lazy.	+		
I am competent and work fast.	0.48		
The plans I make I carry out.	0.52		
*Extraversion* I am usually modest and reserved.	+	0.48	0.64
I am easily motivated and can easily motivate others as well. I tend to be the” strong guy with few words.”	0.68 +		
I am extroverted.	0.57		
*Abolitionism*		0.51	0.71
We must not, under any circumstances, use animals for our own purposes.	0.86		
Custody of farm animals and pets should be abolished, because animals have the right to live in freedom.	0.61		
The use of animals for our own pleasure, such as equestrian sport, dog sports or for the circus, is wrong.	0.51		
*Original Anthropocentrism*		0.56	0.63
Humans are allowed to do whatever they want with animals.	0.46		
We are allowed to treat animals however we want, because they are just animals.	0.50		
We can inflict pain to animals at any time, because they are just animals.	0.30		

^+^indicates omitted items to improve model fit. Model fit: *χ*^2^(149) = 472.56, RMSEA = 0.04, CFI = 0.91, SRMR = 0.04, TLI = 0.88.

Last, descriptive measures indicated that the prevalent gender was female (67%), over male (33%) or diverse (< 0.1%), and ages ranged from 18 to 42 years old (*M* = 21.80, SD = 2.57). On average, participants considered abolitionist ethical concerns related to human-animal relations (mean abolitionism = 3.61) more important than anthropocentric belief (mean original anthropocentrism = 1.26).

### Test of structural models and direct effects

To represent each type of meat, four structural equation models were specified and assessed, controlling for the sociodemographic variables of gender and parental educational level. The researchers analyzed a total of 12 models. The first research objective was to evaluate the relationship between the AEI scale’s domain-specific values (i.e., abolitionism and original anthropocentrism) and differential beef, poultry, and fish consumption. Model 1 therefore specified abolitionism and original anthropocentrism as predictors of varied meat-eating types.^2^ For Model 1a, as seen in Table 2, goodness-of-fit measures were optimal: *χ*^2^(20) = 42.81, RMSEA = 0.03, CFI = 0.98, SRMR = 0.03, TLI = 0.97. Abolitionism (*β* = −0.22, *z* = −3.98_,_ *p* < 0.000) was significantly negatively associated with beef consumption, whereas original anthropocentrism (*β* = 0.23, *z* = 2.90_,_ *p* < 0.001) was significantly positively associated with beef consumption. For Model 1b, as seen in Table 3, goodness-of-fit measures were adequate: *χ*^2^(20) = 38.28, RMSEA = 0.03, CFI = 0.98, SRMR = 0.03, TLI = 0.98. Abolitionism (*β* = −0.22, *z* = −4.04_,_ *p* < 0.000) was significantly negatively associated with poultry consumption, but original anthropocentrism (*β* = 0.04, *z* = 0.49, *p* > 0.05) had no significant association with poultry consumption. Finally, for Model 1c, as seen in Table 4, had adequate fit indices: *χ*^2^(20) = 31.43, RMSEA = 0.02, CFI = 0.99, SRMR = 0.02, TLI = 0.99. Both abolitionism (*β* = −0.08, *z* = −1.64, *p* > 0.05) and original anthropocentrism (*β* = 0.15, *z* = 1.87_,_ *p* > 0.05) were not significantly associated with fish consumption. These results fully support H_1a_ and support H_1b_ for beef consumption alone.

**Table 2:** Beef consumption structural equation models and fit indices, controlling for sex and parental education level.

		Model 1a			Model 2a			Model 3a (Full Mediation)			Model 4a (Partial Mediation)		
Relationship	Hypothesis	*β*	SE	*p*-Values	*β*	SE	*p*-Values	*β*	SE	*p*-Values	*β*	SE	*p*-Values
ABOL → BEEF	H1_a_	−0.22	0.05	0.000^***^				−0.29	0.06	0.000^***^	−0.24	0.07	0.000^***^
ORIG → BEEF	H1_b_	0.23	0.08	0.004^**^				0.23	0.08	0.005^**^	0.23	0.08	0.006^**^
O → BEEF	H2_a_				−0.17	0.08	0.03^*^				−0.17	0.08	0.04^*^
A → BEEF	H2_a_				−0.18	0.09	0.04^*^				−0.07	0.10	0.46
E → BEEF	H2_a_				−0.16	0.18	0.38				−0.20	0.19	0.29
C → BEEF	H2_a_				0.23	0.26	0.36				0.43	0.29	0.13
N → BEEF	H2_a_				−0.05	0.06	0.44				0.03	0.06	0.65
O → ABOL	H3_a_							0.14	0.06	0.03^*^	0.12	0.07	0.08
A → ABOL	H3_a_							0.40	0.08	0.000^*^	0.39	0.08	0.000^***^
E → ABOL	H3_a_							−0.07	0.14	0.63	−0.10	0.15	0.49
C → ABOL	H3_a_							0.29	0.20	0.16	0.36	0.22	0.10
N → ABOL	H3_a_							0.20	0.05	0.000^***^	0.20	0.05	0.000^***^
O → ORIG	H3_b_							−0.05	0.03	0.116	−0.05	0.03	0.16
A → ORIG	H3_b_							−0.17	0.04	0.000^***^	−0.17	0.04	0.000^***^
E → ORIG	H3_b_							0.04	0.07	0.61	0.04	0.07	0.55
C → ORIG	H3_b_							−0.14	0.10	0.16	−0.15	0.10	0.13
N → ORIG	H3_b_							−0.07	0.03	0.007^**^	−0.07	0.03	0.007^**^
													
Model fit indices:													
*χ*^2^ (*df*)		42.81(20)			337.63(99)			475.32(193)			455.04(193)		
RMSEA		0.03			0.05			0.04			0.03		
CFI		0.98			0.92			0.93			0.94		
SRMR		0.03			0.04			0.04			0.04		
TLI		0.97			0.91			0.93			0.93		
													
Δ*χ*^2^(Δ*df*)											20.288 (7)		

^***^p < 0.001, ^**^p < 0.010, ^*^p < 0.050. Beef = beef frequency consumption, O = openness, E = extraversion, C = conscientiousness, A = agreeableness, N = neuroticism, ABOL = abolitionism, ORIG = original anthropomorphism. Beta estimates are standardized.

**Table 3:** Poultry consumption structural equation models and fit indices, controlling for sex and parental education level.

		Model 1b			Model 2b			Model 3b (Full mediation)			Model 4b (Partial mediation)		
Relationship	Hypothesis	*β*	SE	*p*-Values	*β*	SE	*p*-Values	*β*	SE	*p*-Values	*β*	SE	*p*-Values
ABOL → POUL	H1_a_	−0.22	0.06	0.000^***^				−0.23	0.05	0.000^***^	−0.25	0.07	0.000^***^
ORIG → POUL	H1_b_	0.04	0.08	0.63				0.03	0.08	0.68	0.05	0.08	0.58
O → POUL	H2_b_				−0.03	0.08	0.69				−0.03	0.09	0.73
A → POUL	H2_b_				−0.17	0.09	0.05^*^				−0.10	0.10	0.34
E → POUL	H2_b_				−0.30	0.19	0.12				−0.34	0.21	0.96
C → POUL	H2_b_				0.33	0.27	0.22				0.49	0.30	0.10
N → POUL	H2_b_				−0.12	0.06	0.05^*^				−0.06	0.07	0.35
O → ABOL	H3_a_							0.12	0.07	0.07	0.12	0.07	0.09
A → ABOL	H3_a_							0.41	0.08	0.000^***^	0.39	0.08	0.000^***^
E → ABOL	H3_a_							−0.05	0.14	0.70	−0.10	0.15	0.49
C → ABOL	H3_a_							0.30	0.21	0.15	0.36	0.22	0.09
N → ABOL	H3_a_							0.21	0.05	0.000^***^	0.20	0.05	0.000^***^
O → ORIG	H3_b_							−0.05	0.03	0.17	−0.05	0.03	0.17
A → ORIG	H3_b_							−0.17	0.04	0.000^***^	−0.17	0.04	0.000^***^
E → ORIG	H3_b_							0.04	0.07	0.58	0.04	0.07	0.56
C → ORIG	H3_b_							−0.15	0.10	0.13	−0.16	0.10	0.13
N → ORIG	H3_b_							−0.07	0.03	0.006^**^	−0.07	0.03	0.006^**^
													
Model fit indices:													
*X*^2^ (*df*)		38.28(20)			336.40(99)			459.15(200)			450.86(193)		
RMSEA		0.03			0.05			0.03			0.03		
CFI		0.98			0.92			0.94			0.94		
SRMR		0.03			0.04			0.04			0.04		
TLI		0.98			0.91			0.93			0.93		
													
Δ*χ*^2^(Δ*df*)											8.2962(7)		

^***^p < 0.001, ^**^p < 0.010, ^*^p < 0.050. Poultry = poultry frequency consumption, O = openness, E = extraversion, C = conscientiousness, A = agreeableness, N = neuroticism, ABOL = abolitionism, ORIG = original anthropomorphism. Beta estimates are standardized.

**Table 4:** Fish consumption structural equation models and fit indices, controlling for sex and parental education level.

		Model 1c			Model 2c			Model 3c (Full Mediation)			Model 4c (Partial Mediation)		
Relationship	Hypothesis	*β*	SE	p-Values	*β*	SE	p-Values	*β*	SE	p-Values	*β*	SE	p-Values
ABOL → FISH	H1_a_	−0.08	0.05	0.10				−0.05	0.05	0.35	−0.12	0.06	0.06
ORIG → FISH	H1_b_	0.15	0.08	0.06				0.13	0.08	0.10	0.17	0.08	0.034^*^
O → FISH	H2_c_				0.11	0.08	0.16				0.11	0.08	0.17
A → FISH	H2_c_				0.05	0.09	0.59				0.10	0.10	0.29
E → FISH	H2_c_				0.10	0.18	0.59				0.07	0.18	0.70
C → FISH	H2_c_				−0.08	0.26	0.75				0.05	0.26	0.84
N → FISH	H2_c_				−0.04	0.06	0.53				0.01	0.06	0.91
O → ABOL	H3_a_							0.11	0.07	0.10	0.12	0.07	0.09
A → ABOL	H3_a_							0.40	0.08	0.000^***^	0.40	0.08	0.000^***^
E → ABOL	H3_a_							−0.11	0.15	0.50	−0.11	0.15	0.48
C → ABOL	H3_a_							0.38	0.22	0.08	0.38	0.22	0.09
N → ABOL	H3_a_							0.21	0.05	0.000^***^	0.21	0.05	0.000^***^
O → ORIG	H3_b_							−0.04	0.03	0.20	−0.05	0.03	0.17
A → ORIG	H3_b_							−0.17	0.04	0.000^***^	−0.17	0.04	0.000^***^
E → ORIG	H3_b_							0.04	0.07	0.53	0.04	0.07	0.56
C → ORIG	H3_b_							−0.16	0.10	0.12	−0.15	0.10	0.13
N → ORIG	H3_b_							−0.07	0.03	0.006^**^	−0.07	0.03	0.006^**^
													
Model fit indices:													
*X*^2^ (*df*)		31.43(20)			332.17(99)			466.33(200)			439.51(193)		
RMSEA		0.02			0.05			0.03			0.03		
CFI		0.99			0.92			0.93			0.94		
SRMR		0.02			0.05			0.04			0.04		
TLI		0.99			0.92			0.93			0.93		
													
Δ*χ*^2^(Δ*df*)											26.826(7)		

^***^p < 0.001, ^**^p < 0.010, ^*^p < 0.050. Fish = fish frequency consumption, O = openness, E = extraversion, C = conscientiousness, A = agreeableness, N = neuroticism, ABOL = abolitionism, ORIG = original anthropomorphism. Beta estimates are standardized.

The second objective was to investigate the link between the BFPI traits and differential meat consumption patterns. Model 2 assessed the effects of openness, conscientiousness, extraversion, agreeableness, and neuroticism on beef, poultry, and fish consumption frequency. For Model 2a, as seen in Table 2, openness (*β* = −0.174, *z* = −2.19_,_ *p* < 0.010) and agreeableness (*β* = −0.18, z = −2.04_,_ *p* < 0.010) were negatively and significantly related to beef consumption, while neuroticism, extraversion, and conscientiousness were not significantly related to beef consumption. Model fit was adequate: *χ*^2^(99) = 337.63, RMSEA = 0.05, CFI = 0.92, SRMR = 0.04, TLI = 0.91. For Model 2b, as seen in Table 3, neuroticism (*β* = −0.12, *z* = −1.97_,_ *p* < 0.05) and agreeableness (*β* = −0.174, *z* = −1.94_,_ *p* < 0.0.5) had a negative and significant association with poultry consumption. The model had adequate fit indices: *χ*^2^(99) = 336.40, RMSEA = 0.05, CFI = 0.92, SRMR = 0.04, TLI = 0.91. For Model 2c, as seen in Table 4, none of the BFPI traits were significantly associated with fish consumption, with an adequate model fit: *χ*^2^(99) = 332.17, RMSEA = 0.05, CFI = 0.92, SRMR = 0.05, TLI = 0.92. These findings support H_2a_, partially reject H_2b_, and fully reject H_2c_.

### Test of structural equation models and indirect effects

After introducing to the models, the concept of the BFPI traits as precursors of abolitionism and original anthropocentrism, the researchers ran six additional analyses. The first set of analyses assumed BFPI traits had only indirect effects on beef, poultry, and fish consumption *via* abolitionism and original anthropocentrism, constraining the direct paths to equal zero (Models 3a–3c, full mediation). The second set of models allowed all paths to be freely estimated (Models 4a–4c, partial mediation). A Chi-square difference test compared all meat pair-nested models (full mediation vs. partial mediation). Model 4a had significant improved model fit over 3a: Δ*χ*^2^ = 20.28, *p* = 0.001. Model 4b had marginally improved model fit over 3b: Δ*χ*^2^ = 8.29, *p* = 0.030. Model 4c significantly improved model fit over 3c: Δ*χ*^2^ = 26.82, *p* = 0.000. Thus, improved overall fit indices were observed in all three partial mediation models compared to all three full mediation models.

In Model 3a, both abolitionism (*β* = −0.29, *z* = −4.98, *p* < 0.000) and original anthropocentrism (*β* = 0.23, *z* = 2.81, *p* < 0.001) were significant predictors of beef consumption. Similar results were found in Model 4a: Abolitionism (*β* = −0.24, *z* = −3.50, *p* < 0.000) had a significant negative association, while original anthropocentrism (*β* = 0.23, *z* = 2.74, *p* < 0.001) had a significant positive association. In Model 4a, which allowed for the direct effects of BFPI traits on meat consumption, the direct effect of Agreeableness (*β* = −0.07, *z* = −0.74, *p* > 0.45) was attenuated when compared to Model 2a. No other attenuation was found with beef consumption, either for openness (*β* = −0.17, *z* = 0.08, *p* < 0.001) or for other BFPI traits (compare Table 2).

In Model 3b, original anthropocentrism was not associated with poultry consumption (*β* = 0.031, *z* = 0.41, *p* < 0.001), whereas abolitionism (*β* = −0.23, *z* = −4.25, *p* < 0.000) had a significant negative association with poultry consumption. Model 4b also indicated that abolitionism (*β* = −0.25, *z* = −3.51, *p* < 0.000) had a significant negative association with poultry consumption and that original anthropocentrism (*β* = 0.05, *z* = 0.55, *p* > 0.58) had no association with poultry consumption. The direct effects of agreeableness (*β* = −0.10, *z* = −0.96, *p* > 0.34) and neuroticism (*β* = −0.06, *z* = −0.93, *p* > 0.35) were attenuated in Model 4b when compared to Model 2b. Nonsignificant direct effects on poultry consumption thus result for any of the BFPI traits (compare Table 3).

In Model 3c, both abolitionism (*β* = −0.05, *z* = −0.94, *p* > 0.35) and original anthropocentrism (*β* = 0.13, *z* = 1.65*, p* > 0.10) were unrelated to fish consumption. However, Model 4c amplified the relationship of original anthropocentrism (*β* = −0.17, *z* = 2.11, *p* < 0.05) and fish consumption, while abolitionism (*β* = −0.12, *z* = −1.90, *p* > 0.05) remained unrelated. None of the BFPI traits were significantly related with fish consumption in the partial mediation model, which is consistent with Model 2c that only included the direct effects.

The researchers also modeled the effects of the BFPI traits on the AEI scale’s domain-specific values. In Model 3a, openness (*β* = 0.14, *z* = 2.22, *p* < 0.001) was significantly positively associated with abolitionism, whereas in Model 4a, the relationship was only marginally significant (*β* = 0.12, *z* = 1.74, *p* > 0.08). Between Models 3 and 4, both agreeableness (*β* = 0.39, *z* = 4.83, *p* < 0.000) and neuroticism (*β* = 0.20, *z* = 3.84, *p* < 0.000) were significantly positively associated with abolitionism and negatively associated with original anthropocentrism (*β* = −0.17, *z* = −4.36, *p* < 0.000; *β* = −0.07, *z* = −2.71, *p* < 0.001). Extraversion and conscientiousness did not significantly relate to either abolitionism or original anthropocentrism in Models 3 and 4. Since the significant effects of BFPIs on AEI are very similar in Model 3b and Model 4b (see Table 3), as well as in Model 3c and Model 4c (see Table 4), their respective coefficients are not included in this section. These findings, for the most, support H_3a_ and H_3b._

This study’s indirect effects are presented in Table 5. To summarize, four significant indirect effects of BFPI traits *via* abolitionism and original anthropocentrism were established. Agreeableness and neuroticism were associated with less frequent beef consumption indirectly *via* abolitionism and more frequent beef consumption indirectly *via* original anthropocentrism. Two significant indirect effects were established *via* abolitionism on poultry consumption. Low scores in agreeableness and neuroticism were associated with less frequent poultry consumption indirectly *via* stronger abolitionism. As no direct effects were observed in partial mediation model (Model 4b), this result suggests the full mediation of agreeableness (and neuroticism) on poultry consumption *via* animal welfare ethics (Zhao et al., 2010; Rucker et al., 2011). No indirect effects on fish consumption of BFPI traits *via* abolitionism or original anthropocentrism were found, indicating that these domain-specific values are unrelated when fish is measured as the outcome variable. These findings, therefore, partially support H_4a_ and H_4b_.

**Table 5:** Estimates of the indirect effects.

Predictor	Mediator	Meat type	*B*	SE	*Z*	*p*
Openness	ABOL	Beef	−0.03	0.02	−1.68	0.10
Conscientiousness	ABOL	Beef	−0.09	0.06	−1.38	0.17
Extraversion	ABOL	Beef	0.02	0.04	0.66	0.51
Agreeableness	ABOL	Beef	−0.09	0.03	−2.99	**0.003** ^***^
Neuroticism	ABOL	Beef	−0.05	0.02	−2.58	**0.01** ^**^
Openness	ORIG	Beef	−0.01	0.008	−1.31	0.190
Conscientiousness	ORIG	Beef	−0.04	0.029	−1.22	0.22
Extraversion	ORIG	Beef	0.010	0.017	0.56	0.58
Agreeableness	ORIG	Beef	−0.04	0.016	−2.42	**0.02** ^**^
Neuroticism	ORIG	Beef	−0.02	−0.008	−1.93	**0.05** ^*^
Openness	ABOL	Poultry	−0.03	0.026	−1.09	0.27
Conscientiousness	ABOL	Poultry	−0.09	0.082	−1.09	0.28
Extraversion	ABOL	Poultry	0.03	0.050	0.49	0.62
Agreeableness	ABOL	Poultry	−0.10	0.041	−2.37	**0.02** ^**^
Neuroticism	ABOL	Poultry	−0.05	0.023	−2.20	**0.03** ^**^
Openness	ORIG	Poultry	−0.002	0.009	−0.23	0.82
Conscientiousness	ORIG	Poultry	−0.007	0.034	−0.212	0.83
Extraversion	ORIG	Poultry	0.002	0.018	0.10	0.92
Agreeableness	ORIG	Poultry	−0.008	0.022	−0.36	0.72
Neuroticism	ORIG	Poultry	−0.003	0.010	−0.03	0.074
Openness	ABOL	Fish	−0.014	0.017	−0.83	0.41
Conscientiousness	ABOL	Fish	−0.05	0.53	−0.86	0.38
Extraversion	ABOL	Fish	0.01	0.30	0.45	0.65
Agreeableness	ABOL	Fish	−0.05	0.034	−1.39	0.16
Neuroticism	ABOL	Fish	−0.03	0.018	−1.38	0.17
Openness	ORIG	Fish	−0.008	0.012	−0.63	0.52
Conscientiousness	ORIG	Fish	−0.03	0.035	−0.75	0.45
Extraversion	ORIG	Fish	0.007	0.019	0.378	0.70
Agreeableness	ORIG	Fish	−0.03	0.022	−1.34	0.18
Neuroticism	ORIG	Fish	−0.01	0.010	−1.19	0.23

^***^p < 0.001, ^**^p < 0.01, ^*^p < 0.05, B = standardized estimate, SE = standard error, *p* = *p*-values, ABOL = abolitionism, ORIG = original anthropocentrism. Significant indirect effects in bold.

## Discussion

The herein-described study examined the simultaneous role of the BFPI traits and the animal-ethical values of abolitionism and original anthropomorphism in differential meat consumption patterns among a large sample of Chilean undergraduate students. Results were consistent with the hypotheses posed among all structural models.

Model 1a established a significant association of both abolitionism and original anthropocentrism with beef consumption. Less frequent beef consumption was associated with stronger abolitionist values and weaker original anthropocentric values related to the human-animal relationship. More frequent beef consumption was likewise associated with weaker abolitionist values and stronger original anthropocentric values. In the case of poultry with Model 1b, only abolitionist values indicated a significant negative association, while Model 1c revealed no significant relationship of fish consumption either with abolitionism or with original anthropocentrism. This gradual fading of significant associations is in line with prior research in the field (Hellyer et al., 1999; Hölker et al., 2019b; Schobin et al., 2022). Crucial differences between red meat and white meat can explain why different consumption patterns are expected. First, red meat comes from mammals, while white meat comes from birds or sea creatures. These species have very different degrees of phylogenetic relatedness to humans, and phylogenetic relatedness is associated with greater attribution of mental states (Herzog and Shelley, 1997) and empathy (Ingham et al., 2015). Therefore, human-like traits such as sensitivity and sentience or mammalian anthropomorphism seem to play a decisive role in weighting ethical values upon animal species.

In Model 2a, a significant relationship was found between more agreeable and more open individuals and less frequent beef consumption. These results are in consonance with prior literature on the field. No significant relationship was found with other BFPI traits. In Model 2b, more neurotic and more agreeable individuals indicated significantly less frequent poultry consumption. Although not hypothesized, an association between neuroticism and poultry consumption was thus found. In Model 2c, no significant association was found between any BFPI traits and fish consumption, which is in line with prior studies (Schobin et al., 2022). These results further extend previous studies (Keller and Siegrist, 2015; Pfeiler and Egloff, 2018b,c) by providing empirical evidence of the differential antecedent role BFPI traits exert on beef, poultry, and fish consumption and thus offer a broader understanding of these associations as compared to overall meat consumption assessments.

Next, openness, agreeableness, and neuroticism demonstrated significant relationships to abolitionism and to original anthropocentrism.^3^ The directions of relationships were also in accordance with expectations in agreeableness and openness, whereas neuroticism’s unexpected results may offer new explanatory possibilities if confirmed in future research. Agreeableness was positively associated with abolitionism and negatively associated with original anthropocentrism, suggesting that the more agreeable individuals are, the more considerate they are in attributing a high moral-ethical value to animals. Likewise, this suggests that less agreeable individuals are more likely to integrate moral positions into their personal values systems that support or justify animal reification due to human exceptionalism. Similar patterns of association were evident for openness and neuroticism. This is broadly congruent with previous investigations that have examined the relationship between attitudes toward animal welfare and personality traits (Herzog and Mathews, 1997; Hellyer et al., 1999; Furnham et al., 2003; Eckardt Erlanger and Tsytsarev, 2012).

A comparison between the full and partial mediation models lent support for retaining the latter models following a significant Chi-square difference test. Interpretation of Model 4a suggests that neuroticism and agreeableness are associated with differing beef consumption frequency through their positive relationships to abolitionism and negative relationships to original anthropocentrism. Model 4b suggests that agreeableness and neuroticism exert a negative influence on poultry consumption solely *via* abolitionism, whereas nonsignificant effects were found in Model 4c for fish consumption.

In summary, the mediating role of animal-related ethical concerns as domain-specific values helps to explain why certain personality traits are associated with beef and poultry consumption and others are not. The domain-specific approach to animal-ethical concerns in the present study is interpreted as a sub-dimension of the personal value system of individuals and, therefore, as a construct that is correlated with personality traits. Agreeable individuals are characterized by qualities such as empathy and altruism (Hofstee and Goldberg, 1992; Soto and Jackson, 2013) and therefore are more likely to demonstrate benevolent attitudes and exhibit greater affinity for including animal welfare-orientated values in their belief systems. Individuals with higher levels of original anthropocentrism, however, were less agreeable. Personality traits such as empathy or benevolence would not be extensible to other sentient beings in this situation, restricting the ability to ascribe a moral status to other non-human animal species and leading them to consume more animal products.

Although the research did not expect a mediating role between neuroticism and low beef and poultry consumption, the significant results resonate with previous studies related to anticipatory guilt on meat consumption (Wang and Basso, 2019). On the one hand, experimental evidence related to the “meat paradox” has revealed that anthropomorphizing animal meat can alter consumption attitudes and behavioral intentions and induce feelings of guilt (Wang and Basso, 2019; Schobin et al., 2022). On the other, neurotic individuals are characterized as insecure, guilt-ridden, and tense (Hofstee and Goldberg, 1992). In this regard, one way to explain the indirect effects of neuroticism through animal-ethical intuitions on beef and poultry consumption is that individuals with a high degree of neuroticism would tend to base their ethical values about animals, and therefore their human-animal relationship, on feelings of guilt, thus favoring the avoidance or restriction of such food choices. However, more studies are needed to further examine this relationship.

Finally, any effects of personality *via* animal-ethical intuitions disappear for the frequency of fish consumption, which provides additional evidence for a better understanding of the differential meat consumption patterns observed in prior research. As discussed earlier, individuals with high traits of agreeableness and neuroticism might not exhibit tension within their belief and value systems when consuming fish, since they do not experience closeness that invokes empathy or guilt with these types of species.

## Conclusion

The present research considers the mediating role of animal-related ethical values in the association between BFPI traits and different types of meat consumption (i.e., beef, poultry, and fish). Evidence for several antecedent and mediation relationships emerged:

- First, our study adds to the growing evidence base that reveals animal-related ethical values to be an important antecedent of meat consumption.
- Second, our results confirm the findings of previous studies from societies with advanced economies such as (Keller and Siegrist, 2015; Pfeiler and Egloff, 2018c), and (Pfeiler and Egloff, 2020) that the personality traits agreeableness and openness are directly related to meat consumption.
- Third, our results indicate that agreeableness and neuroticism are linked differentially to the consumption of different types of meat through an effect that is mediated by the animal-related ethical values of original anthropocentrism and abolitionism.

By providing the first evidence for the entanglement of personality traits, animal-related ethical values, and meat consumption in an emerging economy context, the current study sheds light on a traditional blind spot in previous studies. It has been argued that religious motives, but also non-Western meat-eating norms, could override the association between personality traits and the frequency and type of meat consumed (Pfeiler and Egloff, 2018b). Considering the intermediary role of animal-related ethical values can, in part, further clarify this argument. This study provides a theoretical mechanism that explains how certain stable personality characteristics and certain domain-specific personal values interact to produce inclinations on the individual level to consume meat. Concretely, our research suggests that more agreeable and more neurotic individuals (and potentially also more open individuals, albeit our evidence is less conclusive in this regard) develop a higher propinquity toward pro-animal-ethical values. These, in turn, are strongly predictive of the consumption of animals that are perceived as human-like. The main takeaway from this is that it would be worthwhile to increase understanding of how personality traits predispose people toward animal-related ethical values and how animal-related ethical positions could be framed better to suit specific personality traits and thus promote dietary change.

## Limitations and further research

Some limitations need to be addressed. Although the results point to a robust relationship with a mediating role of animal-related ethical concerns between personality traits and differential meat consumption, the analysis does not test the causal directions proposed by the mediation model directly. While there are well-documented limitations inherent in testing mediation with cross-sectional data regarding the causality and direction of the effect, decomposing the mediation effect in cross-sectional datasets presents an initial impression of the magnitude of the potential mediation (Preacher, 2015). An extension of the research to longitudinal data or even to experiments that intervene in animal-related ethical values, thus, seems warranted in future studies.

Concerning the type of sample, its characteristics present certain advantages and limitations. Since it corresponds entirely to undergraduate university students, it does not represent a broad subset of the population, so further studies are needed to assess if the results have a more generalizable scope. Nevertheless, this study is one of the first in its field to use participants from the Global South. It contributes to building an evidence base regarding the cultural differences that might underlie individual characteristics affecting the consumption of different types of meat. However, further comparative studies between advanced and emerging economy societies are necessary to elucidate differences or similitudes regarding the mediating effect of animal-related ethical values between personality traits and meat consumption.

Finally, our theoretical framework considers that animal species are attributed ethical values depending on how humans perceive them. While this is in line with previous research explaining that differences in the moral status of different species are mainly due to the affective or anthropomorphic proximity that animal species share with humans (Herzog and Shelley, 1997; Ingham et al., 2015), we did not test this auxiliary hypothesis or include variables that represent this mechanism. The present study and others in the field have, so far, failed to consider how anthropomorphic and affective proximity may interact with underlying personal or cultural factors that influence the acquisition of differential patterns of animal-related moral values. The inclusion of instruments of this type should, therefore, be considered in future research to understand in greater depth the association of animal-related ethics with the human-animal relationship. If included in cross-country comparative research, this would also contribute to a better understanding of the cultural differences that could restrict or favor the consumption of food of animal origin.

## Data availability statement

The original contributions presented in the study are included in the article/supplementary material, further inquiries can be directed to the corresponding author.

## Ethics statement

Ethical review and approval was not required for the study on human participants in accordance with the local legislation and institutional requirements. Written informed consent from the patients/ participants or patients/participants legal guardian/next of kin was not required to participate in this study in accordance with the national legislation and the institutional requirements.

## Author contributions

GH conceived and designed the analysis, collected the data, performed the analysis, and wrote the paper. JS conceived and designed the analysis, collected the data, contributed data or analysis tools, and wrote the paper. AR conceived and designed the analysis and contributed data or analysis tools. All authors contributed to the article and approved the submitted version.

## Funding

This research was supported by the German Federal Ministry of Education and Research grant 01LN1708A.

## Conflict of interest

The authors declare that the research was conducted in the absence of any commercial or financial relationships that could be construed as a potential conflict of interest.

## Publisher’s note

All claims expressed in this article are solely those of the authors and do not necessarily represent those of their affiliated organizations, or those of the publisher, the editors and the reviewers. Any product that may be evaluated in this article, or claim that may be made by its manufacturer, is not guaranteed or endorsed by the publisher.

## Footnotes

^1^Openness describes individuals who tend to appreciate new art, ideas, values, feelings, and creativity (McCrae and John, 1992) and characterizes "the breadth, depth, originality, and complexity of an individual’s mental and experiential life" (John et al., 2008, p. 120). Agreeableness, as Digman (1990) notes, is "a dimension that appears to involve the more humane aspect of humanity—characteristics such altruism, nurturance, caring, and emotional support" (p. 422). Agreeable people are also fair, trustful, and empathic (Goldberg, 1990). Conscientious people tend to behave in organized, dutiful, and responsible ways (Goldberg, 1990; John et al., 2008) and "stay healthier, thrive, and live longer" (Friedman and Kern, 2014, p. 731). Extraversion is defined by the tendency to be talkative, sociable, and to enjoy others’ company (McCrae and John, 1992) and implies "an energetic approach toward the social and material world" (John et al., 2008, p. 120). Finally, neurotic people are insecure, guilt-ridden, and tense individuals (Goldberg, 1990). Neuroticism also relates to frequent negative emotions such as anger, worry, and sadness and to interpersonal sensitivity (McCrae and John, 1992).

^2^Table 2 corresponds to the specifications of the models used to evaluate beef frequency consumption, Table 3 for to the models used to evaluate poultry consumption, and Table 4 to the models used to evaluate fish consumption.

^3^In the case of openness, a significant association was found only in Model 3a, while in Model 4a, the association was attenuated. Both neuroticism and agreeableness had consistent effects in the conceptualized structural models.
